# Parents’ Attitudes About Childhood Vaccines Before and After the COVID-19 Pandemic: A Cross-Sectional Study

**DOI:** 10.3390/medicina61030421

**Published:** 2025-02-27

**Authors:** Vasfiye Demir Pervane, Pakize Gamze Erten Bucaktepe, İsmail Yıldız, Serdar Kardaş, Semih Erdal Tekay, Yıldız Atik, Emel Baran, Mahmut Baran, Tahsin Çelepkolu

**Affiliations:** 1 Department of Family Medicine, Faculty of Medicine, Dicle University, Diyarbakır 21280, Türkiye; gamzebucaktepe@gmail.com (P.G.E.B.);; 2 Department of Biostatistics, Faculty of Medicine, Dicle University, Diyarbakır 21280, Türkiye; 3 Yenişehir District Health Directorate, Diyarbakır 21100, Türkiye

**Keywords:** childhood vaccine attitude, PACV scale, vaccine hesitancy, COVID-19 pandemic

## Abstract

*Background and Objectives*: The COVID-19 pandemic has had a negative impact on immunisation and vaccination attitudes worldwide. The aim of this study was to determine and compare the attitudes of parents towards childhood vaccines by questioning them separately about the periods before and after the COVID-19 pandemic. *Materials and Methods*: This study included parents aged 18–49 years with a child below the age of 5 years who presented at family healthcare centres in a province of Türkiye. The study data form consisted of socio-demographic characteristics and the Parent Attitudes Toward Childhood Vaccines (PACV) scale. An increase in scale scores indicates a negative change in childhood vaccination attitude. *Results*: A total of 1038 parents participated in the study. The results showed that after the pandemic there was an increase in the total and all the subscale points, which was determined to be statistically significant for the total (*p* < 0.001) and safety–efficacy points (*p* < 0.001). Before the pandemic, statistically significantly higher PACV scale points were determined for rural dwellers, the safety–efficacy and total PACV points of females, behaviour and attitude points of those with an education level of primary school or lower, after the pandemic safety–efficacy points of females and those with primary school or lower level of education, and attitude, safety–efficacy, and total scores of those living in rural areas (*p* < 0.05 for all values). The pandemic was seen to have affected the relationship between education level, number of children, and behaviour subscale (*p* = 0.004 and *p* = 0.001, respectively). After the pandemic, there was a statistically significant increase in the mean PACV points of all parents (*p* < 0.001). *Conclusions*: The COVID-19 pandemic has had a negative effect on parents’ attitudes towards childhood vaccines. Efforts should be made to overcome these negative effects to be able to prevent outbreaks of diseases that can be prevented by childhood vaccines.

## 1. Introduction

By preventing millions of deaths each year, vaccines are the most important global success story in the history of public health. Vaccinations are of critical importance in the prevention and control of infectious disease outbreaks [1]. However, despite the significant contributions of vaccination to personal and public health, some parents prefer not to have their children vaccinated. The World Health Organization (WHO) Strategic Advisory Group of Experts on Immunization Working Group defines vaccine hesitancy as “delay in acceptance or refusal of vaccination despite availability of vaccination services” [2]. Vaccine hesitancy has been declared by the WHO to be one of the 10 greatest global threats [3].

Childhood vaccination programs throughout the world were generally adversely affected by the coronavirus disease 2019 (COVID-19) pandemic. There was a decrease in vaccination rates because of the pandemic, access to immunisation services became more difficult, and cases of childhood diseases that are preventable with vaccination increased [4,5,6,7,8]. During the pandemic, vaccinations became a subject widely discussed by the general public, and parental attitudes to childhood vaccinations were investigated. Studies have shown that during the pandemic there was a change in the attitudes of parents to childhood vaccines. Negative attitudes were low in the early periods but increased as the pandemic progressed [9,10]. A positive correlation has been found between knowledge attitude and behaviour to childhood vaccines during the COVID-19 pandemic [11], and it has been shown that gender, education level, and knowledge of and attitudes to childhood vaccines are factors with an impact on parental hesitation [12]. During the pandemic, the attitude to childhood vaccines was found to be effective in the acceptance of the new vaccines; a study in Türkiye on the subject of childhood vaccines showed that parents who were hesitant about childhood vaccines had a more negative attitude towards COVID-19 vaccines [13].

Childhood vaccination in Türkiye is implemented within the scope of the Expanded Immunisation Programme conducted by the Ministry of Health. In this context, childhood vaccines are free and accessible in primary healthcare centres [14]. Although childhood vaccinations are not legally mandatory, families are strongly recommended to be vaccinated and vaccination status is monitored by family physicians. During the COVID-19 pandemic, childhood vaccinations in Türkiye were monitored by family physicians, childhood vaccination rates were kept in the range of 95–99.3% even during periods of restrictions when COVID-19 cases were at their highest, and immunisation services were not interrupted, even in rural areas [15]. In a study conducted during the pandemic in Türkiye, it was stated that the importance of childhood vaccinations was largely accepted by parents, although misinformation about side-effects and safety may trigger anti-vaccine sentiment [16]. Previous studies in Türkiye about the effect of the pandemic on attitudes have also shown that the COVID-19 pandemic negatively affected attitudes towards childhood vaccines [17,18].

Parents’ attitudes towards childhood vaccines are of critical importance for both the well-being of individual children and social health. While a positive approach to vaccination reduces mortality and morbidity rates by protecting children from infectious diseases, it also contributes to the formation of herd immunity in society as a result of high vaccination rates. There is increasing interest in understanding the barriers to vaccination acceptance among vaccine-hesitant parents. The importance of beliefs and attitudes, past experiences, knowledge and awareness, and risk–benefit perception in vaccine acceptance has been shown in previous studies [19]. In research conducted during the pandemic, high confidence in the safety of COVID-19 vaccines and awareness of the necessity of vaccinating children were found to be significantly associated with parents’ intention to vaccinate their children [20]. As the parents have the authority to decide about the vaccination of their child, a positive attitude to vaccines is an important determinant in the decision [21]. Investigations to understand the effect of the pandemic on vaccine confidence and the risk perceptions and behaviours of parents have been recommended, and there are reliable scales predicting the immunization status of children, which can be used to understand the barriers to vaccine acceptance [22,23,24]. Vaccine hesitancy should be taken into consideration in many aspects, and not only children under 5 years of age, but also the perspectives of adolescents on vaccines should be carefully addressed. A systematic review examining vaccine hesitancy in adolescents during the COVID-19 pandemic revealed that safety concerns about vaccines, fear of side effects, belief that the vaccine is unnecessary, and belief in conspiracy theories are among the reasons for avoiding vaccination [25]. On the other hand, although obtaining parental consent in school-based vaccination programmes provides an opportunity to inform families, it can complicate the process. Therefore, the vaccine consent process needs to be improved through more effective communication strategies, electronic systems and training programmes [26]. Within the scope of the goals of increasing vaccination rates in Italy, in the case of parental refusal of vaccination, vaccination is made possible if the adolescent has the desire to be vaccinated, and in this case promises to make a positive contribution to raising awareness about vaccination among adolescents who have not yet entered adulthood [27]. All these findings highlight the need for multifaceted strategies to reduce hesitancy towards vaccination among adolescents and parents.

As the emergence of the COVID-19 pandemic was not foreseen, there are few studies in the literature that have compared attitudes to vaccinations before and after the pandemic and evaluated the changes [9,18,28]. Considering the negative impact of the COVID-19 pandemic on childhood vaccination rates [4,5,6,7,8], it has become very important to determine the change in parents’ attitudes towards childhood vaccines caused by the COVID-19 pandemic in order to prevent outbreaks of vaccine-preventable childhood diseases. The hypothesis of this study was that parents’ attitudes towards childhood vaccinations became more negative after the COVID-19 pandemic compared to before the pandemic. The aim of this study was to determine and compare the attitudes of parents with children under the age of five years towards childhood vaccines by questioning them separately about the periods before and after the COVID-19 pandemic, and to evaluate the relationship with sociodemographic characteristics.

## 2. Materials and Methods

### 2.1. Study Design, Setting, and Participants

This cross-sectional, analytical study was prepared in accordance with the Strengthening the Reporting of Observational Studies in Epidemiology (STROBE) guidelines [29]. Approval for the study was granted by the Non-Interventional Research Ethics Committee of Dicle University (decision no: 96, dated: 14 April 2022) and the necessary institutional permission was obtained. This study was conducted in compliance with the Helsinki Declaration.

This study included parents aged 18–49 years with a child below the age of 5 years who presented for any reason at 175 family healthcare centres in the province of Diyarbakır, located in the south-east of Türkiye. The sample size was calculated using G*Power 3.1.9.7 software. The number of subjects to be included in the study was found to be at least 327 with a power of 0.95, effect size of 0.2, and α error probability of 0.05 by a priori analysis. From an initial total of 1163 parents participating in the study, 125 questionnaires were excluded from the analyses due to incomplete data. Therefore, the data of 1038 parents were analysed. A post hoc power analysis indicated that the sample size used in this study had sufficient power for the proposed analysis.

The study data were collected using a questionnaire administered between July and September 2022. Those who wished to participate face-to-face provided written informed consent and the participants who wished to complete the questionnaire online provided an online consent form. The researchers invited parents to participate in the study at the family healthcare centres where the researchers worked, and visits were made to other family healthcare centres to invite parents to participate. All the participants were fully informed about the study.

The study inclusion criteria were defined as age 18–49 years, with a child aged < 5 years, no mental incapacity that would prevent participation, and voluntary participation in the research. The study exclusion criteria were defined as age < 18 years or >49 years, not having a child or child not aged < 5 years, or not willing to participate in the study. In Türkiye, it is recommended by the Ministry of Health that childhood vaccinations are completed by the age of 48 months (except for the booster dose of tetanus vaccine at the age of 13 years) [14]. In order to both retrospectively question the vaccination attitude before the pandemic and to facilitate recall, parents with children under 5 years of age were selected. As the experiences of older parents may be different because of their age and it may be difficult to recall information, the age range of parents to be included in the study was limited to 18–49 years, which is accepted as the adult fertility age range.

### 2.2. Measures

The study data form consisted of two sections. Items in the first section questioned the subject’s age, gender, marital status, education level, number of children, children aged < 5 years, and history of childbirth after 11 March 2020 (the date of the first recorded COVID-19 case in Türkiye). The second section of the form consisted of the Parent Attitudes Toward Childhood Vaccines (PACV) scale [30]. Each question on the scale was asked twice for separate responses related to before and after the pandemic.

The PACV scale, developed by Opel et al., is a valid and reliable scale that evaluates the attitudes of parents to childhood vaccinations [30]. Validity and reliability studies of the Turkish version of the scale were conducted by Çevik et al., and the Cronbach alpha value of the scale was 0.676, and the test–retest results were good (internal consistency coefficient: 0.93, *p* = 0.001) [31]. The scale consists of 15 items in 3 subscales of behaviour, attitude, and safety and efficacy. Items 1 and 2 of the scale refer to behaviour, items 3–6 and 11–15 refer to attitude, and items 7–10 measure safety and efficacy. The responses to items 1, 2, and 11 are in the form of yes, no, I do not know, items 3 and 15 are scored from 0–10 points, items 4–7, 13, and 14 have 5 response options ranging from “strongly agree” to strongly disagree”, items 8–10 from “not at all concerned” to “very concerned”, and item 12 from “not at all hesitant” to “very hesitant”. The PACV scale was selected to evaluate parental hesitancy because it is understandable, easy to complete, and has been proven to be a valid and reliable scale for Turkish society, with cut-off scores for the sub-dimensions of attitude, behaviour, and safety and efficacy, which determine the presence of vaccine hesitancy.

In the scoring of the scale, responses indicating hesitancy are scored with 2 points, those showing no hesitancy with 0 points, and indecision with 1 point. The total scale points range from 0 to 30. The total raw points are re-calculated using a simple linear calculation method for items with missing data, to fit a scale of values ranging from 0 to 100, and thus a converted score is obtained. A converted PACV score of <50 indicates no vaccination hesitancy and scores ≥ 50 show vaccination hesitancy. An increase in points in the total, converted, and all subscales of the scale shows a negative change in the vaccination hesitancy of parents towards childhood vaccinations.

The Cronbach’s alpha value of the PACV scale used in our study was calculated as 0.739 for pre-pandemic and 0.747 for post-pandemic.

### 2.3. Statistical Analysis

Data obtained in the study were analysed statistically using IBM SPSS for Windows vn. 26.0 software. Quantitative data were stated as mean ± standard deviation (SD) values, and correlation coefficients (r), and categorical variables were presented as number (n) and percentage (%). Conformity of the data to normal distribution was assessed with the Kolmogorov–Smirnov test, histogram, and skewness and kurtosis tests. There were determined to be no significant deviations from normal distribution. The change in scale points from before to after the pandemic was examined with the Paired Samples *t*-test, and the vaccine hesitation status was compared with the rate of responses given to each scale item using the McNemar test. The effect of the pandemic on sociodemographic characteristics was analysed with a General Linear Model and Repeated Measurements Variance Analysis. Relationships between variables were examined with a Pearson correlation analysis. The scale total and subscale points were compared with the sociodemographic variables using the Independent Samples *t*-test in two groups, and when there were more than two groups, variance homogeneity was evaluated with the Levene test, then with One-way ANOVA if variances were homogenous and with Welch ANOVA if not. The Pearson chi-square (χ2) test was used in the comparisons of the presence or absence of vaccination hesitation and sociodemographic characteristics. Hypotheses were taken as two-tailed and a value of *p* < 0.05 was accepted as statistically significant.

### 2.4. Ethics Approval and Consent to Participate

This study was conducted according to the guidelines of the Declaration of Helsinki and was approved by the Non-Interventional Research Ethics Committee of Dicle University (decision no: 96, dated: 14 April 2022). The study questionnaire was designed so that the participant had to first read the explanations about the study and provide informed consent before starting to complete the questionnaire.

## 3. Results

### 3.1. Sociodemographic Data

A total of 1038 parents participated in this study and the study participants comprised 77.2% females and 22.8% males with a mean age of 32.2 years, 98.8% were married, 60.5% had one or two children, and the mean number of children was 2.5. The place of residence was reported to be central districts by 89.3% of the participants and 60.6% of the parents had given birth to a child after the onset of the pandemic (Table 1).

### 3.2. PACV Scale Items and Responses

In the responses given referring to after the pandemic, there was seen to be a statistically significant increase in the beliefs that there was a probability that one of the vaccinations was not safe, that the child could have a serious side-effect from the vaccination, and that the vaccination may not provide protection against the disease (*p* < 0.001 for all) (Table 2). Although following the recommended vaccination program was thought to be a good idea by a high rate of the parents after the pandemic (*p* = 0.001), the parents evaluated themselves as more hesitant about childhood vaccinations after the pandemic (*p* < 0.001), and if they had a child today, they would be less willing for that child to have all the recommended vaccinations (*p* = 0.029). After the pandemic, a higher rate of parents stated that most diseases prevented by vaccines are serious diseases (*p* < 0.001) and a lower rate stated that it was better to acquire immunity through illness than through vaccination (*p* = 0.001). Compared to before the pandemic, a lower rate of parents delayed or did not have their child vaccinated after the pandemic. The rate of parents who reported not delaying childhood vaccinations was 82.8% before the pandemic and this increased to 99.2% after the pandemic. It was observed that after the pandemic parents trusted the information received about vaccinations less (*p* = 0.016) and a higher rate thought that children had more vaccinations than were necessary (*p* < 0.001).

### 3.3. PACV Scale Scores

The total and subscale (behaviour, attitude, and safety and efficacy) points obtained from the PACV scale by the parents were calculated separately for before and after the pandemic, and then compared (Table 3).

There was determined to be an increase in all the scale points of the parents after the pandemic, and this increase showed a negative change. The increase in the behaviour and attitude points of the parents did not create a statistically significant difference (*p* = 0.349 and *p* = 0.489, respectively). The safety and efficacy total and converted points increased and these increases were determined to be statistically significant (*p* < 0.001 for all).

### 3.4. Relationships Between the PACV Scale Scores and Sociodemographic Characteristics

The relationships were examined between sociodemographic characteristics and all the subscales of the PACV scale before and after the COVID-19 pandemic (Table 4).

The total points obtained from the PACV scale and the safety and efficacy points were determined to be statistically significantly higher for females than males (*p* < 0.05 for all). The effect of the pandemic variable on the relationship between gender and the behaviour, attitude, safety and efficacy, and total points was examined, and the pandemic was not determined to have made a statistically significant difference (*p* = 0.896, *p* = 0.292, *p* = 0.593, and *p* = 0.702, respectively).

In the comparisons based on education level, it was seen that the behaviour and attitude towards childhood vaccinations before the pandemic were more negative in the study participants with an education level of primary school or lower compared to those with a higher level of education (*p* = 0.029 and *p* = 0.031, respectively). After the pandemic, there was seen to be an increase in the safety and efficacy points of the participants at all education levels, showing a statistically significant change between the education level and the safety and efficacy subscale points (*p* = 0.039). The pandemic variable was determined to have a significant effect only on the behaviour subscale in the relationship between education and the PACV subscales (*p* = 0.004), and there was no effect on the attitude, safety and efficacy, and total points (*p* = 0.524, *p* = 0.565, and *p* = 0.912, respectively).

In the evaluation according to place of residence, the behaviour, attitude, safety and efficacy, and total points of the PACV scale were determined to be significantly higher for the parents living in rural areas compared to those in urban areas before the pandemic (*p* < 0.05 for all). After the pandemic, there was a decrease in the behaviour points of the parents living in rural areas, which eliminated the significant difference (*p* = 0.151). The attitude, safety and efficacy, and total points of the parents living in rural areas remained significantly higher than those of the parents living in urban areas after the pandemic (*p* < 0.05 for all). The variable of the pandemic was not determined to make a difference to the behaviour, attitude, safety and efficacy, and total points in the relationship between place of residence and PACV subscales (*p* = 0.153, *p* = 0.795, *p* = 0.279, and *p* = 0.205, respectively).

In the evaluation made on the basis of the number of children, no significant difference was determined before and after the pandemic in the behaviour, attitude, safety and efficacy, and total points according to the number of children (*p* > 0.05 for all). After the pandemic, there was seen to be a decrease in the behaviour points of the parents with five or more children, and an increase was determined in the points of the parents with four or fewer children. The pandemic caused a significant change only in the behaviour dimension in the relationship between the number of children and the PACV subscales (*p* = 0.001), and there was not found to be any effect on the relationship with the attitude, safety and efficacy, and total points (*p* = 0.323, *p* = 0.973, and *p* = 0.627, respectively).

The results of the parents with and without children after the onset of the pandemic were compared with the PACV subscale and total points, and no significant relationship was determined with having a new infant both before and after the pandemic (*p* ≥ 0.05 for all). The pandemic variable was not determined to have a significant effect on the relationship between the status of having a child and the total points of the PACV scale and behaviour, attitude, and safety and efficacy subscale points (*p* = 0.599, *p* = 0.237, *p* = 0.335, and *p* = 0.546, respectively).

Correlations between age and the number of children are presented in Table 5.

Both before and after the pandemic, there was determined to be a negative correlation between age and attitude, safety and efficacy, and total scale points. As age increased, there was observed to be a positive change in attitude to childhood vaccinations. No statistically significant difference was determined in the correlation between the number of children and the PACV scale points.

### 3.5. PACV Scale—Vaccine Hesitation

The study participants were categorised as having vaccine hesitation present in those with a PACV score of ≥50 and absent in those with a score <50. The rate of vaccine hesitation increased from 17.2% before the pandemic to 17.9% after the pandemic (Table 6).

**Figure 1:**
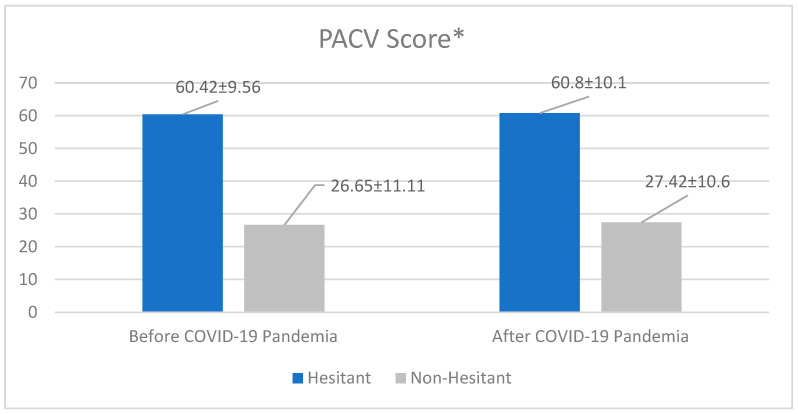
Distribution of mean PACV scores according to vaccine hesitation. * Mean ± SD: Average, SD.

The mean PACV score of the parents with vaccine hesitation was 60.42 ± 9.56 before the pandemic and this increased to 60.8 ± 10.1 after the pandemic (Figure 1).

Although the increase in the rate of vaccine hesitation from before to after the pandemic was not statistically significant (*p* = 0.457), the increase from before to after the pandemic in mean PACV points of both the parents with and without vaccine hesitation was determined to be statistically significant (*p* < 0.001 for both).

The presence or absence of vaccine hesitation before and after the COVID-19 pandemic was compared with sociodemographic characteristics (Table 7).

Before the pandemic, vaccine hesitation was determined at the rate of 27% in those living in rural areas and at 16.1% in urban dwellers (*p* = 0.004). After the pandemic, these rates decreased to 25.2% for those living in rural areas, and increased to 17% for those in urban areas. There continued to be a statistically significant relationship between place of residence and vaccine hesitation (*p* = 0.034).

## 4. Discussion

In this study that investigated the effect of the COVID-19 pandemic on parents’ attitudes to childhood vaccinations, the results demonstrated a negative change after the pandemic compared to the period before, especially in the sub-dimensions of the safety and effectiveness of vaccines. Gender, education level, place of residence, and number of children were found to be factors associated with the attitudes of parents to childhood vaccination. In addition, gender, education level, place of residence, and number of children were determined to be significant variables in the effect of the pandemic on the attitudes of parents to childhood vaccinations. There are studies in the literature that have investigated the attitudes to childhood vaccinations and vaccine hesitancy during and after the pandemic [9,18,28,32]; however, the current study is distinguished by the fact of having investigated and compared the attitudes to childhood vaccination in a specific population before and after the pandemic separately and having shown the effect of the pandemic on the attitude to vaccination and sub-dimensions thereof.

Of the parents in this study, 77.2% (n = 80) were female. In previous studies that have evaluated the vaccine hesitancy of parents, it has been seen that the participants comprised many more females [21,33,34,35,36]. The participants in a previous study in Türkiye were also seen to be female at a similar rate (72.1%, n = 770) [13]. The current study participants were selected from parents with a child aged < 5 years who presented at a family healthcare centre. As the family healthcare centres in Türkiye provide immunisation services and monitor pregnancies, infants, and children, many more women present at these outpatient clinics, and this can be considered an important factor in the greater participation of females in this study. Considering that women are more likely to apply to primary health care services, planning the studies to be carried out for the development of positive vaccination attitudes to include women as the target group may yield efficient results.

The study results showed that the total points obtained by the parents from the PACV scale, and the points obtained from the scale sub-dimensions (behaviour, attitude, and safety–efficacy) increased after the pandemic compared to before the pandemic. Although these increases reflected a negative change in vaccination attitude, the increase in safety–efficacy, total, and converted scores also created a statistically significant difference. In parallel with the current research, other studies have also shown a negative change in the attitude to vaccination after the COVID-19 pandemic. Previous studies using the PACV scale have reported that the pre-pandemic mean converted points were in the range of 20.5–28.5 [22,32,37,38], and studies after the pandemic have shown these to be higher in the range of 30.96–64.28 [13,17,31,39]. In the current study, the total converted points obtained from the PACV scale were mean 32.47 before the pandemic and increased to 33.4 after the pandemic. The pre-pandemic attitude to vaccination scores of the parents were thus found to be higher than the scores reported in the literature. When it is considered that the data were collected in a period after the onset of the pandemic, which was accepted as a time when the pandemic was officially continuing, the negative attitude to vaccination after the pandemic could be a reason that the pre-pandemic attitude was interpreted or remembered as negative. A previous study in Türkiye reported that the COVID-19 pandemic had a negative effect on the attitudes to childhood vaccinations of 79.15% of parents [17]. The attitude to vaccination is multifactorial and variability can be seen according to sociodemographic characteristics, countries, and even periods of the pandemic. A study that was conducted in the USA after the onset of the COVID-19 pandemic investigated the change in attitude to childhood vaccinations using the PACV short scale, and reported that a negative attitude showed fluctuations during the pandemic, as lower in the early periods and increasing in the later periods [9]. Another study in the USA using the vaccine hesitancy scale evaluated the effect of the pandemic on childhood vaccination hesitancy, and there was seen to be a significant increase in the hesitancy of parents during the pandemic compared to before the pandemic [33], The WHO vaccine hesitancy scale was used in a study in Türkiye investigating the vaccine hesitancy of parents in the first and second peak periods of the pandemic, and there was reported to be an increase in the vaccine hesitancy scale points in the second peak compared to the first peak, reflecting a negative change [18]. The PACV sub-scale dimension points of behaviour, attitude, safety–efficacy before the pandemic were seen to be lower in a study conducted in Malaysia compared to the points of the parents in the current study [34]. In another Turkish study conducted after the pandemic, the total and subscale points of behaviour, attitude, and safety–efficacy were found to be higher than the points of the current study, with the exception of the pre-pandemic safety–efficacy points [39]. As the COVID-19 pandemic could not be foreseen, no study was found that evaluated the vaccine attitude of the population after the pandemic, in which parental vaccination attitudes were evaluated before the pandemic. In order to evaluate the effect of the pandemic variable in the current study, the same scale was completed twice, taking into account before and after the pandemic, and the pre-pandemic attitude was compared with studies conducted before the pandemic, and the post-pandemic attitude was compared with studies conducted after the pandemic. The fact that pre-pandemic attitudes were questioned retrospectively in the current study may have caused recall bias.

The attitude of the parents to vaccination and vaccine hesitancy is an important marker of the intention to have the child vaccinated, and parents who are hesitant may have their children vaccinated less or may delay vaccinations more [13,21,40,41]. Therefore, more interest should be shown to parents who are hesitant and the attitudes of parents to vaccinations should be well evaluated. In a study before the pandemic, in which parents from 18 European countries participated, 24% were reported to be somewhat hesitant, and 4% very hesitant [42]; whereas a study in the USA showed that 30.4% of parents were very or somewhat hesitant [22]. In a study conducted in Albania after the pandemic, 5% of parents evaluated themselves as very hesitant and 12% as very hesitant [35]. A study evaluating the attitudes to childhood vaccinations of a Turkish population found that those who were more hesitant and those who did not have their children vaccinated had higher vaccine hesitancy points [43]. In the current study, vaccine hesitancy increased from 17.2% before the pandemic to 17.9% after the pandemic. Although this increase was not found to be statistically significant, the increases in mean PACV points of those with and without vaccine hesitancy were determined to be statistically significant. Using the PACV scale, studies have shown pre-pandemic vaccine hesitancy in the range of 7.7–25% [21,22,36,37], and that these rates increased to 9.38–58.71% after the pandemic [13,17,39]. In contrast, a study in the USA found no difference in vaccine hesitancy during the pandemic compared to the pre-pandemic period, showed vaccine hesitancy at the rate of 24%, and while the COVID-19 pandemic was not associated with changes in vaccine hesitancy of parents, there were seen to be changes in the trust felt about information received about vaccines [28]. Another study in the USA showed that generally there was an increase in childhood vaccine hesitancy after the pandemic compared to before, and although there was no change in vaccine confidence, there was an increase in the perceived risk [33]. While high levels of hesitancy lead to low vaccine demand, low levels of hesitancy do not necessarily mean high vaccine demand [2]. It must be taken into consideration that the attitudes and hesitancy of parents towards vaccination and subsequently vaccination behaviour are complex and can differ according to sociodemographic characteristics, geography, and time and periods of pandemics, and should therefore not be dealt with as a single dimension.

Gender, age, education level, and economic status are the factors most associated with vaccine hesitancy [11,12,41,43,44,45]. In a meta-analysis that evaluated parental willingness to vaccinate their children during the pandemic, gender, age, and the stage of the pandemic were reported to be the most important determinants [12]. Studies have shown differences in the relationship between vaccine hesitancy and gender. In studies conducted before and after the COVID-19 pandemic, greater vaccine hesitancy has been reported in females [41,43]. Although a systematic review after the pandemic showed greater vaccine hesitancy in females [45], there are also studies reporting that vaccine hesitancy after the pandemic was greater in males or that there was no relationship with gender [10,13,46]. In the current study, females had a more negative attitude to vaccination than males both before and after the pandemic, this attitude became more negative after the pandemic, and the dimensions of safety and efficacy of childhood vaccinations were seen to be more negatively affected. A review that evaluated vaccine acceptance during the pandemic showed a negative correlation between female gender and the intention to vaccinate [45]. Considering the role of the mother in the healthcare of her child, it is important to develop and maintain positive vaccination attitudes of mothers during periods such as epidemics or pandemics.

The current study results showed that as the age of parents decreased, negative attitudes increased both before and after the pandemic, and the attitudes to the safety and efficacy of vaccines became more negative after the pandemic. Being a young parent has been associated with a greater number of days that the child is under-immunized [13]. A study conducted before the pandemic in Malaysia reported greater vaccine hesitancy in young parents [34]. There are studies in literature showing lower levels of vaccine hesitancy in males over the age of 50 after the pandemic [43] and there are also studies that have shown no relationship between age and attitudes to vaccination [13]. There are also studies showing a greater tendency of the elderly population towards vaccination [45]. A study in the USA associated decreased risk perception with the age range of 45–54 years after the pandemic compared to before [33], while a study in Türkiye reported that parents aged < 35 years had a more negative attitude to the safety and efficacy of vaccines [46]. The fact that people in the younger age group use social media more or have easier access to information may cause them to be exposed to more anti-vaccine stimuli, which may be related to higher vaccine hesitancy in this group. Individuals with vaccine hesitancy should be contacted, taking into account the characteristics of their age.

The current study results demonstrated that vaccine hesitancy was higher in those with an education level below that of primary school and the hesitancy rates fell after the pandemic, whereas the rates of vaccine hesitancy increased for parents with a higher level of education. While the behaviour and attitude of parents with education below primary school level was more negative before the pandemic, this was seen to change in a positive direction after the pandemic, but there was a negative change in the dimension of safety. Furthermore, the current study showed that there was an effect of the pandemic on the relationship between education level and behaviour. Education level is an important factor in vaccine acceptance and hesitancy, and variability can be seen according to countries and time periods. In a study that included parents from 18 European countries, the hesitancy scores of parents with an education level of high school and below were found to be higher [42]. Studies in Türkiye during the pandemic reported significantly higher vaccine hesitancy in parents with an education level of middle school and below, and in comparisons of parents who rejected or did not reject vaccinations, there were seen to be more university graduates in those who rejected vaccinations [31,47]. However, in a study in the USA, having a master’s degree was associated with decreased risk perception after the pandemic compared to before [33].

In another review that analysed the factors affecting vaccine acceptance and hesitancy during the pandemic, education level and employment were determined to be the two most important factors in the vaccination of individuals. A low education level and low income were associated with low vaccination rates and were negatively correlated with the intention to vaccinate [45]. When lockdowns were most intense during the COVID-19 pandemic in India, children aged <1 year were seen to be vaccinated less, and this decrease was greater in poor families with low education levels, and the greatest difference was due to education [48]. Another study conducted during the pandemic reported that the children of illiterate females were under-immunized 5-fold more than the children of educated females [49]. A study conducted after the pandemic in Türkiye reported that the education level of mothers was observed to have a significant effect on having children vaccinated at the right time [50], while another study found no association between education level and vaccine hesitancy, although the more hesitant group were seen to be educated to university level or above [13]. No relationship was determined between education level and monthly income in a study in Malaysia before the pandemic [34], while a study in the USA showed that those with an education level of higher than university were more hesitant [41]. There are also studies conducted after the pandemic that have shown no relationship with education level [13,46].

The presence of vaccine hesitancy was evaluated according to the place of residence in the current study, and although there was a slight increase in the hesitancy rate of urban dwellers (16.1% to 17%), and the rates of rural dwellers fell (27% to 25.2%), the rate of hesitancy of rural dwellers was found to be significantly higher both before and after the pandemic. In addition, all the sub-dimensions of the scale were negative before the pandemic for those living in rural areas and there were positive changes in behaviour after the pandemic, whereas more negative changes were seen for those living in urban areas. There was reported to be a correlation between the place of residence and vaccine hesitancy in a study conducted during the pandemic, and the vaccine hesitancy was seen to be greater in those living in rural areas [28]. It has been shown that most of the individuals showing vaccine refusal and hesitancy were born in developed geographical regions of Türkiye and have high income and education levels [51]. The fact that the current study results showed a decrease in vaccine hesitancy and positive changes in behaviour in rural dwellers could be because those living in rural areas and especially in villages were less affected by the intense restrictions and lockdowns of the pandemic and the media discussions about vaccinations, and that those in rural areas have a lower level of education. In our findings, a decrease in vaccine hesitation rates and positive changes in behaviour were observed in rural areas after the pandemic. This may be due to the fact that rural people were less affected by the intense closures and restrictions during the pandemic, that those living in rural areas, especially in villages, were less exposed to intense media discussions on vaccines due to limitations in internet access, and that rural people read less and try to access information online due to their lower education level. Vaccine hesitancy can become a major global health issue, negatively influenced by anti-vaccination campaigns on social media platforms, and ultimately a lack of knowledge or misinformation about vaccines can influence people’s decision to get vaccinated [45]. It has been reported that 50% of parents of children under 5 years of age in the UK have been exposed to negative messages about vaccines on social media [52]. The negative impact of social media platforms on vaccine hesitancy in Asian countries during the pandemic has been demonstrated, with reliance on social media platforms for vaccine-related information being associated with a higher likelihood of COVID-19 vaccine hesitancy. Moreover, this has been reported not only for COVID-19 vaccines but also for childhood vaccines [53]. Cross-sectional studies reporting the relationship between social media addiction and vaccination intentions have mostly observed a negative relationship [54]. Social media is a powerful tool for the dissemination of misinformation, and the increase in misinformation and disinformation of unknown origin during the pandemic has led to the need for major social media platforms to impose restrictions on anti-vaccine and unreliable sources of information [55]. It has been reported that individual attitudes towards vaccination are based on a rational assessment of the situation and that the only important factor positively affecting vaccination intention is scientific knowledge [56]. Increasing media literacy, health authorities being more active on social media, and developing better algorithms to filter misinformation can be used to combat misinformation and media-induced narratives [55]. In a study in Türkiye, it is reported that parents living in rural areas had less information about non-routine vaccinations than those living in urban areas, but no relationship was determined between support for non-vaccination and place of residence [57]. A study in Indonesia which evaluated the relationship of an urban–rural difference with the immunisation status of children reported that urban immunisation coverage was greater than rural, and this difference could be attributed to regional cultural factors affecting compliance with health guidelines, the level of concern related to infection, and differences in socioeconomic status [58]. During the COVID-19 pandemic in Türkiye, childhood vaccinations were followed up by family doctors, and it was seen that the rates of childhood vaccinations remained between 95% and 99.3% even during the lockdown periods when there were the highest numbers of COVID-19 cases, and immunisation services were not interrupted in rural regions [15].

In the current study, although no relationship was determined between the number of children in the family and vaccine hesitancy, the pandemic was observed to affect the relationship between the number of children and behaviour. In a cohort study that evaluated the change in childhood vaccination hesitancy of mothers, it was seen that as the age of the children increased, the hesitancy of the mothers decreased, and mothers with other older children were less hesitant than first-time mothers [38]. Higher vaccination rates have been reported before the pandemic for children with three or more siblings [59], and not having the child vaccinated on time after the pandemic has been associated with there being two or more children in the family [7]. A study conducted in Malaysia before the pandemic showed greater vaccine hesitancy in parents with fewer than two children [34], and a study in Ireland reported no relationship of the presence of childhood vaccine hesitancy with education level and the number of children [32]. A study conducted after the pandemic in Türkiye reported that the behaviour and general attitude of parents with three or more children were more negative [46] and another study reported greater vaccine hesitancy in those with no children [43].

Despite its practical contributions, this study also has some limitations. Although this study was not conducted in a single centre, the results cannot be interpreted nationally or globally because the data were collected from a single province. This study was cross-sectional and self-reported in design, which could have led to recall bias. The participants were asked retrospectively about their pre-pandemic attitudes. In this case, it is a limitation that the pre-pandemic attitude is remembered retrospectively, or the pre-pandemic attitude is remembered under the influence of the post-pandemic attitude. New vaccine studies for COVID-19 were conducted during the pandemic period, and rumours about the safety of these vaccines and participants’ exposure to incorrect vaccine information can be considered as potential confounding factors in evaluating the impact of the pandemic on vaccine attitudes. Vaccination attitude is a complex multi-faceted concept that can be influenced by instant events, media shares, or misinformation and can change negatively in a short time. In this context, considering that parents’ post-pandemic vaccine attitude was evaluated in 2023, parents’ perceptions of vaccine-related health issues may have evolved and may no longer reflect the current reality in 2025. Considering the positive contributions of immunisation to public health, this situation leads to the necessity to evaluate vaccine attitude in the target groups to be examined repeatedly over the years. It should not be forgotten that media influence, which was not questioned in this study, may also have had an effect on changes in vaccines attitude. These limitations and confounding factors may have reduced the effectiveness of this study.

## 5. Conclusions

In conclusion, the results of this study demonstrated that the COVID-19 pandemic had a negative effect on the attitudes of parents to childhood vaccinations, in the sub-dimensions, the attitudes to the safety and efficacy of childhood vaccinations were especially negatively affected, and that sociodemographic characteristics were important variables on the parental attitudes to childhood vaccinations. The attitude to vaccinations is multifactorial and can show variability according to sociodemographic characteristics, countries, cultures, time, pandemics, and even different periods of pandemic. In order not to lose the gains acquired to date in childhood vaccinations, and to not experience childhood disease pandemics that can be prevented by vaccines, it is important that the negative effects of the COVID-19 pandemic are dealt with in all aspects by identifying the characteristics of parents, and that every effort is made to overcome these effects.

In addition to quantitative studies, qualitative research is needed to evaluate the negative impact of the pandemic more comprehensively and reveal the problems in detail. The impact of the pandemic on attitudes towards childhood immunisations should be determined on a regional basis and training or intervention strategies should be developed according to the defined characteristics of the regions. It should not be ignored that anxiety and speculation about the COVID-19 vaccines developed during the pandemic period may also have a negative impact on the attitude towards the safety and effectiveness of childhood vaccines, and parents should be listened to, informed, and reassured at the point of identifying concerns. The impact of online information networks on society should not be ignored, and online networks, especially social media, should be used to alleviate negative effects.

## Figures and Tables

**Table 1:** Sociodemographic Characteristics of the Study Participants.

		n (1038)	%
Average Age (Years)		32.2 ± 6.1 (18–49)	
Average Number of Children		2.5 ± 1.5 (1–11)	
Gender	Female	801	77.2
	Male	237	22.8
Education	Literate/Primary	296	28.5
	Secondary/High School	385	37.1
	University	357	34.4
Marital Status	Married	1026	98.8
	Single	12	1.2
Residency	Urban	927	89.3
	Rural	111	10.7
Number of Children	1–2	628	60.5
	3–4	297	28.6
	≥5	113	10.9
Child Birth During Pandemic *	Yes	629	60.6
	No	409	39.4

* After 11 March 2020 (the date of the first reported COVID-19 case in Turkey).

**Table 2:** PACV Scale Items and Responses for Before and After the COVID-19 Pandemic.

		COVID-19 Pandemic				
		Before (n = 1038)		After (n = 1038)		*p* *
		n	%	n	%	
Have you ever delayed having your child get a shot for reasons other than illness or allergy?	Yes	82	7.9	81	7.8	NA **
	Do not know	97	9.3	0	0	
	No	859	82.8	957	92.2	
2. Have you ever decided not to have your child get a shot for reasons other than illness or allergy?	Yes	58	5.6	69	6.6	NA **
	Do not know	57	5.5	0	0	
	No	923	88.9	969	93.4	
3. How sure are you that following the recommended shot schedule is a good idea for your child?	0–5	176	17	164	15.8	0.001
	6–7	174	16.8	155	14.9	
	8–10	688	66.3	719	69.3	
4. Children get more shots than are good for them.	Agree	161	15.5	172	16.6	<0.001
	Not sure	267	25.7	220	21.2	
	Disagree	610	58.8	646	62.2	
5. I believe that many of the illnesses that vaccines prevent are severe.	Agree	702	67.6	732	70.5	<0.001
	Not sure	192	18.5	162	15.6	
	Disagree	144	13.9	144	13.9	
6. It is better for my child to develop immunity by getting sick than to get a shot.	Agree	163	15.7	155	14.9	0.001
	Not sure	202	19.5	179	17.2	
	Disagree	673	64.8	704	67.8	
7. It is better for children to get fewer vaccines at the same time.	Agree	355	34.2	369	35.5	0.106
	Not sure	335	32.3	317	30.5	
	Disagree	348	33.5	352	33.9	
8. How concerned are you that your child might have a serious side effect from a shot?	Concerned	732	70.5	803	77.4	<0.001
	Not sure	97	9.3	76	7.3	
	Non-concerned	209	20.1	159	15.3	
9. How concerned are you that any one of the childhood shots might not be safe?	Concerned	736	70.9	813	78.3	<0.001
	Not sure	95	9.2	75	7.2	
	Non-concerned	207	19.9	150	14.5	
10. How concerned are you that a shot might not prevent the disease?	Concerned	731	70.4	777	74.9	<0.001
	Not sure	107	10.3	90	8.7	
	Non-concerned	200	19.3	171	16.5	
11. If you had another infant today, would you want him/her to get all the recommended shots?	Yes	850	81.9	834	80.3	0.029
	Do not know	112	10.8	111	10.7	
	No	76	7.3	93	9	
12. Overall, how hesitant about childhood shots would you consider yourself to be?	Hesitant	211	20.3	262	25.2	<0.001
	Not sure	121	11.7	120	11.6	
	Non-hesitant	706	68	656	63.2	
13. I trust the information I receive about shots.	Agree	774	74.6	747	72	0.016
	Not sure	180	17.3	200	19.3	
	Disagree	84	8.1	91	8.8	
14. I am able to openly discuss my concerns about shots with my child’s doctor.	Agree	799	77	806	77.6	0.311
	Not sure	148	14.3	135	13	
	Disagree	91	8.8	97	9.3	
15. All things considered, how much do you trust your child’s doctor?	0–5	112	10.8	114	11	0.108
	6–7	134	12.9	133	12.8	
	8–10	792	76.3	791	76.2	

* Mc Nemar, ** Not Applicable.

**Table 3:** Comparison of Scale Scores Before and After the COVID-19 Pandemic.

PACV Scale Scores	Before the Pandemic	After the Pandemic	Difference	95% CI of the Difference	*p* *
	Mean ± SD	Mean ± SD	Mean ± SD		
Behaviour	0.27 ± 0.86	0.29 ± 0.90	−0.019 ± 0.663	−0.060–0.021	0.349
General Attitudes	3.82 ± 3.78	3.85 ± 3.90	−0.032 ± 1.481	−0.122–0.058	0.489
Safety and Efficacy	5.53 ± 2.20	5.86 ± 2.02	−0.328 ± 1.294	−0.406–−0.249	<0.001
Total	9.62 ± 4.94	10.0 ± 4.97	−0.379 ± 2.078	−0.505–−0.252	<0.001
Converted	32.47 ± 16.76	33.40 ± 16.61	0.929 ± 7.073	−0.1360–−0.498	<0.001

* Paired test.

**Table 4:** Comparison of Sociodemographic Data with PACV Scale Scores Before and After the COVID-19 Pandemic.

		Before the COVID-19 Pandemic								After the COVID-19 Pandemic							
		Behaviour		General Attitudes		Safety and Efficacy		Total		Behaviour		General Attitudes		Safety and Efficacy		Total	
		Mean ± SD	*p*	Mean ± SD	*p*	Mean ± SD	*p*	Mean ± SD	*p*	Mean ± SD	*p*	Mean ± SD	*p*	Mean ± SD	*p*	Mean ± SD	*p* *^,^**
Gender	Female	0.3 ± 0.9	0.610	3.8 ± 3.8	0.929	5.7 ± 2.2	<0.01	9.8 ± 4.9	0.049	0.3 ± 0.9	0.198	3.9 ± 3.9	0.528	6.0 ± 1.9	<0.01	10.2 ± 4.9	0.025
	Male	0.2 ± 0.8		3.8 ± 3.8		5.0 ± 2.3		9.1 ± 5.0		0.2 ± 0.8		3.7 ± 3.8		5.4 ± 2.2		9.4 ± 5.1	
Education	Literate/ Primary	0.4 ± 1.0	0.029	4.1 ± 3.6	0.031	5.6 ± 2.2	0.824	10.0 ± 4.7	0.060	0.3 ± 0.9	0.104	3.8 ± 3.6	0.585	6.0 ± 2.0	0.039	10.1 ± 4.6	0.404
	Secondary/ High School	0.2 ± 0.8		4.0 ± 3.7		5.6 ± 2.2		9.8 ± 4.6		0.2 ± 0.8		4.0 ± 3.9		6.0 ± 1.9		10.2 ± 4.7	
	University	0.3 ± 0.8		3.4 ± 3.9		5.5 ± 2.2		9.1 ± 5.4		0.4 ± 1.0		3.7 ± 4.1		5.6 ± 2.1		9.7 ± 5.6	
Residency	Urban	0.3 ± 0.8	0.036	3.6 ± 3.7	<0.01	5.4 ± 2.2	<0.01	9.3 ± 4.9	<0.01	0.3 ± 0.9	0.151	3.7 ± 3.8	<0.01	5.8 ± 2.1	<0.01	9.8 ± 5.0	<0.01
	Rural	0.5 ± 1.0		5.3 ± 4.0		6.4 ± 1.5		12.1 ± 4.7		0.4 ± 1.0		5.2 ± 4.0		6.4 ± 1.6		12.1 ± 4.6	
Number of Children	1–2	0.27 ± 0.85	0.893	3.89 ± 3.86	0.652	5.61 ± 2.16	0.125	9.77 ± 4.99	0.470	0.29 ± 0.89	0.370	4.02 ± 3.95	0.214	5.85 ± 1.98	0.928	10.16 ± 5.04	0.377
	3–4	0.26 ± 0.83		3.65 ± 3.61		5.5 ± 2.21		9.41 ± 4.80		0.32 ± 0.94		3.61 ± 3.72		5.90 ± 2.05		9.82 ± 4.85	
	≥5	0.3 ± 0.93		3.88 ± 3.60		5.16 ± 2.34		9.35 ± 4.95		0.19 ± 0.75		3.54 ± 3.73		5.82 ± 2.06		9.56 ± 4.81	
Child Birth During the Pandemic	Yes	0.25 ± 0.84	0.388	3.74 ± 3.79	0.406	5.61 ± 2.18	0.179	9.60 ± 4.87	0.852	0.29 ± 0.89	0.899	3.71 ± 3.82	0.138	5.88 ± 1.99	0.648	9.88 ± 4.86	0.321
	No	0.30 ± 0.88		3.94 ± 3.73		5.42 ± 2.21		9.66 ± 5.03		0.29 ± 0.90		4.07 ± 3.93		5.82 ± 2.04		10.1 ± 5.12	

* Independent Samples *t* test, ** One-way ANOVA or Welch ANOVA.

**Table 5:** Correlation Coefficients of Age and Number of Children with PACV Scale Scores.

PACV Scale Scores	Pandemic	Age ^a^	Number of Children ^a^
Behaviour	Before	−0.014	0.006
	After	−0.036	−0.022
General Attitudes	Before	−0.078 *	−0.002
	After	−0.079 *	−0.048
Safety and Efficacy	Before	−0.074 *	−0.041
	After	−0.093 **	0.026
Converted	Before	−0.100 **	−0.012
	After	−0.114 **	−0.033
Total	Before	−0.095 **	−0.019
	After	−0.105 **	−0.030

**^a^** Pearson correlation. * Correlation is significant at the 0.05 level. ** Correlation is significant at the 0.01 level.

**Table 6:** Comparison of Hesitancy Rates Before and After the COVID-19 Pandemic.

		After the COVID-19 Pandemic			
		Non-Hesitant	Hesitant	n (%)	*p* *
Before the COVID-19 Pandemic	Non-Hesitant	823	36	859 (82.8)	0.457
	Hesitant	29	150	179 (17.2)	
	n (%)	852 (82.1)	186 (17.9)	1038 (100)	

* Mc Nemar Test.

**Table 7:** Comparisons of Vaccine Hesitation with Sociodemographic Characteristics Before and After the COVID-19 Pandemic.

		Vaccine Hesitation						
		Before the Pandemic			After the Pandemic			Total n (%)
		Non-Hesitant n (%)	Hesitant n (%)	*p* *	Non-Hesitant n (%)	Hesitant n (%)	*p* *	
Gender	Female	654 (81.6)	147 (18.4)	0.083	649 (81.0)	152 (19.0)	0.103	801 (100.0)
	Male	205 (86.5)	32 (13.5)		203 (85.7)	34 (14.3)		237 (100.0)
Education	Literate/ Primary	238 (80.4)	58 (19.6)	0.369	243 (82.1)	53 (17.9)	0.982	296 (100.0)
	Secondary/ High School	319 (82.9)	66 (17.1)		315 (81.8)	70 (18.2)		385 (100.0)
	University	302 (84.6)	55 (15.4)		294 (82.4)	63 (17.6)		357 (100.0)
Residency	Urban	778 (83.9)	149 (16.1)	0.004	769 (83.0)	158 (17.0)	0.034	927 (100.0)
	Rural	81 (73.0)	30 (27.0)		83 (74.8)	28 (25.2)		111 (100.0)
Number of Children	1–2	511 (81.4)	117 (18.6)	0.343	508 (80.9)	120 (19.1)	0.382	628 (100.0)
	3–4	252 (84.8)	45 (15.2)		247 (83.2)	50 (816.8)		297 (100.0)
	≥5	96 (85.0)	17 (15.0)		97 (85.8)	16 (14.2)		113 (100.0)
Child Birth During the Pandemic	Yes	521 (85.5)	108(14.5)	0.937	520 (82.6)	109 (17.3)	0.539	629 (100.0)
	No	338 (82.6)	71 (17.3)		332 (81.1)	77 (18.8)		409 (100.0)
Total	n (%)	859 (82.8)	179 (17.2)		852 (82.1)	186 (17.9)		1038 (100.0)

* Chi-square test.

## Data Availability

The data of this study are available from the corresponding author upon reasonable request.
